# Social and Metabolic Characteristics Associated With Multiple DKA Admissions at a Large County Hospital

**DOI:** 10.1210/jendso/bvad173

**Published:** 2024-01-19

**Authors:** Josh Peedikayil, Shrenika Reddy, Rohit Nair, Uma Gunasekaran, Carolyn Nelson, Musa Shakoor, Zahid Ahmad

**Affiliations:** UT Southwestern Medical School, Dallas, TX 75390, USA; Department of Internal Medicine, Division of Endocrinology, Diabetes and Metabolism, Mercy Clinic, Festus, MO 63028, USA; UT Southwestern Medical School, Dallas, TX 75390, USA; Department of Internal Medicine, Division of Endocrinology, UT Southwestern Medical Center, Dallas, TX 75390, USA; St. Joseph's/Candler Physician Network–Endocrinology, Savannah, GA 31405, USA; Department of Internal Medicine, VA North Texas, Dallas, TX 75216, USA; Department of Internal Medicine, Division of Endocrinology, UT Southwestern Medical Center, Dallas, TX 75390, USA

**Keywords:** diabetic ketoacidosis, type 1 diabetes mellitus, substance use, hospital admissions, safety-net hospital

## Abstract

**Context:**

Diabetic ketoacidosis (DKA) is a preventable, deadly, and costly complication of type 1 diabetes mellitus (T1DM). Some individuals with T1DM have recurrent DKA admissions.

**Objective:**

We sought to characterize social factors that differ between patients with single vs multiple DKA admissions at an urban, safety-net hospital.

**Methods:**

We queried the electronic health records for T1DM patients admitted for DKA from 2019 to 2021. Admission laboratory values, demographic information, and detailed social histories were collected and analyzed statistically, including logistical regression.

**Results:**

A total of 243 patients were admitted for DKA, 64 of whom had multiple DKA admissions. There was no significant difference between the groups in their admission laboratory values, hospital length of stay, health-care payer status, history of homelessness, current employment, living alone, independence of activities of daily living, and barriers to discharge. T1DM patients with multiple DKA admissions had greater rates of substance use disorder (33.0% vs 60.9%; *P* < .001), especially with cannabis (6.7% vs 25.0%; *P* < .001), tobacco (26.3% vs 46.3%; *P* = .002), and psychoactive substance use (1.1% vs 6.3%; *P* = .043). Regression models of substance use showed increased risk with any substance use (odds ratio [CI] 3.17 [1.78-5.73]; *P* < .001) and cannabis (3.70 [1.55-8.83]; *P* = .003).

**Conclusion:**

We identified substance use as a possible predictor of T1DM patients at risk for multiple DKA admissions. Our findings identify a group of T1DM patients for whom interventions may help to decrease recurrence of DKA episodes within similar community hospital populations.

In the absence of exogenous insulin or in the presence of precipitating illnesses, patients with type 1 diabetes (T1DM) can develop diabetic ketoacidosis (DKA), the most serious diabetic emergency that carries a high morbidity and mortality burden. DKA, in the overwhelming majority of cases, is a preventable, deadly, and costly complication of T1DM.

The number of admissions with DKA in the United States is more than 130 000 per year and accounts for an estimated total cost of $2.4 billion annually [1]. The importance of psychological factors in the incidence of DKA has been highlighted in recent studies [2, 3]. Through retrospective analysis, we sought to better characterize social factors that are different between patients with single vs multiple admissions at an urban safety-net hospital.

## Materials and Methods

### Setting and Participants

The study was approved by the UT Southwestern Institutional Review Board. Patient data were extracted from the electronic health records (EHR) at Parkland Memorial Hospital in Dallas, Texas, a large-volume center that serves as the safety-net community hospital for patients throughout the North Texas area. The EHR at Parkland is Epic Systems Corporation [4].

### Data Collection

We queried the EHR for patients admitted to Parkland Hospital from January 1, 2019 to December 31, 2020, with DKA. DKA status was determined by the documented final International Classification of Diseases, Tenth Revision (ICD-10) diagnosis at discharge, a strategy with 90% positive predictive value to identify cases of DKA [5]. Multiple studies have used this methodology for determining DKA diagnoses, both as a primary outcome for clinical trials, as well as for documenting safety efficacy [6, 7].

The charts were then manually reviewed to collect information including demographic information, psychiatric diagnoses, social/psychological factors, and laboratory data about their individual admissions. We used the first encounter to evaluate variables predicting DKA readmission. The first serum laboratory values including glucose, calculated bicarbonate (HCO_3_), CO_2_, pH, β-hydroxybutyrate, lipid panel, and glycated hemoglobin A_1c_ taken at the time of admission were collected. Social/psychological factors were collected from standardized social worker documentation at each patient's first DKA admission. This standardized documentation included information about living arrangements, history of homelessness, incarceration, barriers to discharge, and independence of activities of daily living (ADL). Furthermore, the documentation included diagnosis of substance use disorder, including alcohol, tobacco, opiate, cannabis, and psychoactive drugs. Patients who were using multiple substances were included in the variable count for each substance, and not placed in their own group.

All charts meeting inclusion criteria were reviewed by J.P., M.S., and S.R., with supervision by a University of Texas Southwestern (UTSW) endocrinologist, Z.A. The study was approved by the UTSW Institutional Review Board with a waiver of access to personal health information (in the form of EHR data) without written consent from the participants. Once patient information was obtained from the EHR, it was deidentified and stored (encrypted) within the Research Electronic Data Capture (RedCap) database for all statistical analysis and data dissemination.

### Statistical Analysis

Statistical analysis was performed using SPSS version 17. Descriptive statistics were summarized using median and interquartile range for continuous variables and number and percentages for dichotomous variables, and summary statistics are presented in tabular form. For dichotomous variables, chi-square analysis was performed. For continuous variables, *t* tests were performed for Gaussian variables, and for non-Gaussian continuous variables, nonparametric testing with Wilcoxon rank sum tests were used. Variables with count data were treated as continuous for statistical analysis. Multivariable logistic regression was performed for further assessment of association and determination of odds ratios (ORs). This regression model included patients’ individual substance use diagnoses (alcohol, tobacco, cannabis, opiate, cocaine, psychoactive). A univariate logistic regression was performed to evaluate association and ORs for patients within the category of having any substance use diagnosis. This was done with the aim of delineating which individual substances would be the most predictive. A *P* value of .05 was used for consideration of statistical significance.

## Results

### Demographic Information and Past Medical History

We identified 243 unique patients with at least 1 admission to Parkland Memorial Hospital with a diagnosis of DKA. Of those, 64 patients were admitted 2 or more times with DKA. The median number of admissions among these 64 was 2 (interquartile range, 2-4). Baseline characteristics and demographics between the 2 groups, single and multiple admissions, were similar, (Table 1). The mean (SD) age for the group was 34.0 (11.6) years, with a BMI of 25.1 (5.9), with a male predominance (57.8%) and self-identified as White (53.5%) or Black (44.9%). The number of DKA admissions per patient ranged from 1 to 29 admissions. Of the 64 patients with multiple admissions, 38 (60%) were admitted exactly twice and 59 (92%) had 2 to 5 admissions.

**Table 1.** Demographic and admission laboratory values of studied patients

Characteristic	Total	Single admits	Multiple admits	*P*
No.	243	179	64	—
Age, y*^a^*	34.0 ± 11.6	34.4 ± 11.3	32.5 ± 12.4	.123
BMI, mean*^a^*	25.1 ± 5.9	25.5 ± 5.9	24.0 ± 5.5	.069
Length of stay, d*^a^*	3.26 ± 2.65	3.25 ± 2.73	3.29 ± 3.07	.846
History of homelessness, %	21	20.1	23.4	.264
History of incarceration, %	17.7	14.5	26.6	.007
Current employment, %	33.3	31.8	37.5	.504
Living alone, %	16.0	16.2	15.6	.939
History of schizophrenia (%)	2.1	1.7	3.1	.609
Sex, n female (%)	104 (42.8)	70 (39.1)	34 (53.1)	.052
Race, n (%)				
White	130 (53.5)	99 (55.3)	31 (48.4)	.627
Black	109 (44.9)	77 (43.0)	32 (50.0)	
Other*^b^*	4 (1.6)	3 (1.7)	1 (1.6)	
Ethnicity, n (%)				
Hispanic	80 (32.9)	60 (33.5)	20 (31.3)	.7459
Method of payment, n (%)				
Charity	90 (37.0)	65 (36.3)	25 (39.0)	.833
Commercial	25 (10.3)	20 (11.2)	5 (7.8)	
Government	47 (19.3)	35 (19.6)	12 (18.8)	
Self-Pay	81 (33.3)	59 (33.0)	22 (34.4)	
Laboratory values				
HbA_1c_, %	11.7 ± 2.4	11.7 ± 2.4	11.7 ± 2.3	.911
Glucose*^a^*, mg/dL	519 (332)	516 (301.5)	494 (237.75)	.466
HCO_3_, mEq/L	14.9 ± 7.0	15.1 ± 7.5	14.2 ± 5.5	.302
PCO_2_, mm Hg	33.6 ± 10.0	34.0 ± 10.7	32.2 ± 7.6	.148
pH	7.2 ± 0.13	7.2 ± 0.13	7.2 ± 0.11	.814
BHB mg/dL	6.34 ± 3.3	6.29 ± 3.5	6.44 ± 2.9	.781

Shown for the total patient population, as well as separated between those with single vs multiple diabetic ketoacidosis admissions.

Abbreviations: BHB, β-hydroxybutyrate; BMI, body mass index; HbA_1c_, glycated hemoglobin A_1c_; HCO_3_, calculated bicarbonate; PCO_2_, partial carbon dioxide pressure.

^*a*^Nonparametric testing used for analysis; values displayed as median (interquartile range).

^*b*^Other races included Asian and Native American.

There was no significant difference between the groups in regard to their history of homelessness (21%; *P* = .264), current employment (33.3%; *P* = .504), or living alone (16%; *P* = .939). Patients with multiple DKA admissions had a significantly greater rate of incarceration history than those with only a single admission (26.6% vs 14.5%; *P* = .007) (see Table 1).

Overall, 2.1% of patients had a history of schizophrenia. The rate of this diagnosis was not significantly different between the 2 groups.

Most patients were uninsured and relied on charity funding (37.0%) or self-pay (33.3%). Government payment through either Medicare or Medicaid accounted for 19.3% of patients and the remaining 10.3% had commercial insurance (see Table 1).

### Admission Laboratory Values

There were no differences between the 2 groups regarding their diagnostic diabetes and DKA laboratory values. Mean (SD) values were serum glucose of 549 mg/dL (243 mg/dL), HCO_3_ 14.9 mEql/L (7.0 mEql/L), partial carbon dioxide pressure (PCO_2_) 33.6 mm Hg (10 mm Hg), pH 7.2 (0.13), β-hydroxybutyrate 6.34 mg/dL (3.3 mg/dL), and hemoglobin A_1c_ 11.7% (2.4). There was no statistical difference between the hospital length of stay between the 2 groups (3.25 days vs 3.29; *P* = .846). Of the 243 patients meeting inclusion criteria, 84 also had serum lipids drawn at the time of admission (Table 2). There was no difference (mean, SD) between total cholesterol levels (194 mg/dL, 66), low-density lipoprotein cholesterol (102 mg/dL, 49), or serum triglycerides (270 mg/dL, 333). Serum high-density lipoprotein cholesterol (HDL-C), however, did differ between the groups (*P* = .011). Those with a single admission had a mean (SD) HDL-C of 43 mg/dL (14) vs 55 mg/dL (23) in those with multiple admissions.

**Table 2.** Admission lipid laboratory values of studied patients

Characteristic	Total	Single admits	Multiple admits	*P*
No.	83	59	24	—
Total cholesterol, mg/dL	181.5 (59)	187 (83.5)	179 (66)	.604
LDL-C, mg/dL	91 (51.5)	90 (54.25)	91 (45)	.226
HDL-C, mg/dL	44.5 (21)	42 (15.5)	50 (25)	.011
Triglycerides, mg/dL	144 (143)	172 (223)	132 (79)	.135

Shown for the total patient population, as well as separated between those with single vs multiple diabetic ketoacidosis admissions. Values displayed as median (interquartile range).

Abbreviations: HDL-C, high-density lipoprotein cholesterol; LDL-C, low-density lipoprotein cholesterol.

### Barriers to Discharge and Activities of Daily Living Independence

The most common barrier to discharge documented on all patients was medical stability (37.9%), followed by financial stability (26.3%) and follow-up (19.3%) (Table 3). There were no significant differences between the rates of each barrier to discharge or total discharge barriers. Patients both with single and multiple admissions had similar barriers to discharge.

**Table 3.** Documented barriers to discharge and activities of daily living of the studied patients

Characteristic	Total	Single admits	Multiple admits	*P*
Barriers to discharge				
Follow-up,	19.3	17.9	23.4	.334
Financial, %	26.3	26.8	25.0	.777
Medical stability, %	37.9	37.4	39.1	.817
Transport, %	6.2	5.0	9.4	.232
Medication, %	7.8	7.3	9.4	.589
Housing, %	4.9	5.0	4.7	≥.999
Safety, %	1.2	0.6	3.1	.170
Education, %	2.1	2.2	1.6	≥.999
Patient choice, %	1.2	1.7	0	.568
Any discharge barriers, %	59.3	60.3	56.3	.568
ADL independence				
Feeding,	91.4	91.1	92.2	.783
Bathing, %	90.5	89.4	93.8	.306
Toileting, %	90.5	89.9	92.2	.599
Driving, %	44.4	44.1	45.3	.871
Finance, %	49.8	48.0	54.7	.362
Independent of all ADL, %	37.0	35.8	40.6	.489

Shown as percentages of the total population as well as separated between those with single vs multiple diabetic ketoacidosis admissions.

Abbreviation: ADL, activities of daily living.

Regarding ADL, almost all patients were independently able to perform feeding (91.4%), bathing (90.5%), and toileting (90.5%) without any assistance. A total of 44.4% of patients were able to independently drive and 49.8% were able to handle finances. There were no significant differences between either group of patients regarding independence of any individual ADL or total independent ADL.

### Substance Use

**Figure 1.**
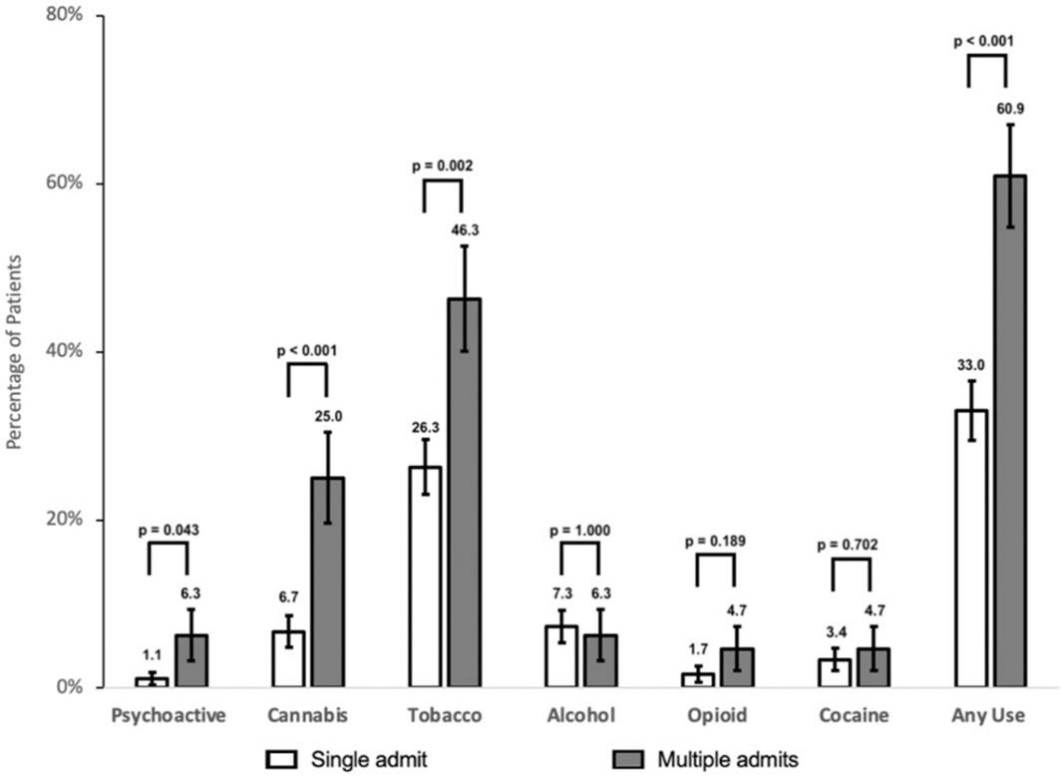
Substance use between patients, separated between those with single and multiple diabetic ketoacidosis admissions. Bars represent the percentage of patients using each substance, with values above their respective bars. SE bars are presented for each sample population, and *P* values are shown for all differences.

In the total patient population, 40.3% of patients had at least 1 diagnosis of substance use disorder from one of the aforementioned substances. Patients with multiple admissions had a significantly increased rate of substance use overall compared to those with a single admission (33.0% vs 60.9%; *P* < .001) (Fig. 1). Specifically, patients with multiple admissions had a higher rate of tobacco use (26.3% vs 46.3%; *P* = .002), cannabis use (6.7% vs 25.0%; *P* < .001), and psychoactive substance use (1.1% vs 6.3%; *P* = .043) than those with a single admission. All other substances, including alcohol, opioid, and cocaine, showed no difference between usage rates in the 2 study groups. A univariate regression model showed increased DKA readmission odds in individuals with any substance use (*P* < .001; OR [95% CI] 3.17 [1.78-5.73]) (Table 4). Using a multivariable regression model for use of individual substances, cannabis use remained independently associated with a significantly increased odds of DKA readmission (*P* = .003; OR [95% CI] 3.70 [1.55-8.83]).

**Table 4.** Documented substance use disorder diagnosis among the patient population

Substance use	Odds ratio	*P*
Alcohol, %*^a^*	0.84 (0.25-2.86)	.779
Tobacco, %*^a^*	1.88 (0.97-3.66)	.062
Opioid, %*^a^*	2.81 (0.49-16.09)	.245
Cannabis, %*^a^*	3.70 (1.55-8.83)	.003
Cocaine, %*^a^*	0.43 (0.08-2.31)	.324
Psychoactive, %*^a^*	4.81 (0.73-31.88)	.104
Any substance use, %*^b^*	3.17 (1.78-5.73)	<.001

Results from multivariable and univariate analysis.

Presented as odds ratio (95% CI) and *P* values.

^*a*^Multivariable analysis of these groups.

^*b*^Univariate analysis used.

## Discussion

Our findings describe multiple psychosocial factors that are significantly more prevalent in patients with T1DM who are readmitted with DKA. Those with recurrent DKA were more likely to have been previously incarcerated. While there has been much research looking at the increased risk of DKA and hyperosmolar hyperglycemic syndrome in patients who are in prison, few studies have looked at these events in those following release [8]. Randall et al [1], in their 2011 study of 164 minority patients with DKA at a similar large community hospital, did not find a difference between those with a single vs multiple DKA episodes. However, they similarly did see a significant increase in incarceration history as the number of admissions increased.

Many studies have examined substance use and substance use disorder among patients readmitted with DKA episodes; however, few have looked at individual substances to determine differences among those with recurrent DKA [1, 2, 9]. Substance use was common among our cohort of patients. Namely, cannabis use was significantly increased in those with multiple DKA admissions. Findings from the T1DM Exchange Clinic registry have recently shown an increase in prevalence of cannabis use among T1DM patients who are admitted for DKA (not necessarily multiple DKA admission), and our findings build on this association by identifying cannabis use as independently associated with DKA readmission [10]. Possible etiologies of this association including alteration of gut motility or consequences of cannabis hyperemesis syndrome have been proposed; however, further research needs to be completed to better understand this relation [11, 12].

An increased incidence of tobacco use in those with T1DM who are hospitalized for DKA compared to controls without DKA was seen in studies such as by Hamblin et al [9]; however, comparison between single-episode DKA vs recurrence was not addressed. Our study showed an increased incidence of tobacco use in those with DKA readmission based on 2-sample *t* tests; however, the multivariable analysis did not show an independent association. Few prior studies, in a similar fashion to ours, have compared patients with single and multiple DKA admissions. Some, such as Hare et al [13], in their study of 128 patients at an urban center in Australia, found an increased prevalence of tobacco usage among those with multiple admissions. Others, such as Michaelis et al [14], who also performed a similar study, found no difference in tobacco usage between the groups.

Regarding other substances, many studies have looked at substance use, but few have individually looked at substances to see which are more associated with increased risk for readmission [1, 13]. Based on multivariable analysis of these substances, only cannabis use showed an independent association. Use of psychoactive substances, which showed a significant difference in the chi-square analysis, did not have an independent association on the multivariable analysis. An association between psychoactive substances and DKA readmission has not been characterized in previous studies. We also show similarity to Modzelewski et al [15], who did not see an increase in DKA admissions in those that used cocaine.

A lipid panel is typically not a standard laboratory test that is run on patients admitted for DKA. Likely for that reason, only 83 patients admitted for DKA had a lipid test drawn throughout their admission. However, there was a significant increase in the level of HDL-C in those with readmissions for DKA. The association between T1DM and HDL-C level has been explored in multiple studies; however, none have shown an association between HDL-C levels and incidence of DKA or recurrent DKA in this population [16-18]. Our findings are unlikely to be related to selection bias among individuals who had lipid measurements taken, as there were no demographic differences between those with and without lipid measurements. Mechanistically, HDL is involved in multiple pathways beyond lipid metabolism, including inflammation, endothelial function, cognition, hormone transport, and kidney function. As understanding of HDL's various functions continues to improve, a plausible explanation for our findings may emerge.

### Limitations

There are several limitations to this study. First, our study is limited to a single center over a limited time period, so our findings may not extrapolate to all other community health-care settings over longer periods. Our data also came from documented ICD-10 codes within patient charts, so classification of data could be dependent on individual providers. Some studies, such as Davis and Davis [19], have seen inaccuracies up to 36% in ICD-10 diagnosis codes of DKA, although that study was performed in Australia, where diagnostic guidelines and practices may be different from those of our US-based study. Based on laboratory data (see Table 1), some admissions may not meet widely accepted diagnostic DKA criteria of pH less than 7.30 and HCO_3_ less than 18 mmol/L. These findings imply that patients may have been partially treated prior to laboratory analyses. Patients with T1DM are often given intravenous fluids and insulin based on point-of-care fingerstick glucose levels and provider discretion. This would alter the results of the first set of formal laboratory data. However, this does not alter the main outcomes from this study. Use of any substance was not evaluated biochemically. Instead, ICD-10 diagnosis codes were used as determinants of substance use. Next, the study only looked at patients admitted for DKA during our specified time period, defining recurrence as 2 or more admissions within that time. This is a method similar to the studies conducted by Lyerla et al [3] and Bradford et al [2]. Admittedly, we cannot determine if these were an inaugural presentation of DKA as it is possible that patients may get care from other hospital systems. The study also cannot describe precipitants of DKA in this population; as it was not systematically documented in the EHR, we could not comment on those factors. Our study can also only evaluate associations and cannot establish a causative relation, though it is possible that one exists. A future, interventional study would be needed to help establish cause and effect. Further, another study across multiple, similar large community hospitals in different areas would provide more consensus about how psychosocial factors affect patients with T1DM in this population.

Future work can build on this new knowledge by using implementation science methods targeted toward individuals with T1DM with tobacco and cannabis use. Such methods should include patient perspectives on what interventions would best improve treatment compliance and reduce DKA recurrence.

## Conclusions

Our study discovered significant associations between readmission with DKA and multiple social and behavior factors within patients with T1DM, especially regarding substance use and incarceration. Our findings identify a group of T1DM patients for whom interventions may help to decrease recurrence of DKA episodes within similar community hospital populations.

## Data Availability

Restrictions apply to the availability of some or all data generated or analyzed during this study to preserve patient confidentiality or because they were used under license. The corresponding author will on request detail the restrictions and any conditions under which access to some data may be provided.

## Abbreviations

ADL: activities of daily living
DKA: diabetic ketoacidosis
EHR: electronic health records
HCO_3_: calculated bicarbonate
HDL-C: high-density lipoprotein cholesterol
ICD-10: International Classification of Diseases, Tenth Revision
PCO_2_: partial carbon dioxide pressure
T1DM: type 1 diabetes mellitus
